# High-throughput analysis of tissue microarrays using automated desorption electrospray ionization mass spectrometry

**DOI:** 10.1038/s41598-022-22924-4

**Published:** 2022-11-07

**Authors:** Nicolás M. Morato, Hannah Marie Brown, Diogo Garcia, Erik H. Middlebrooks, Mark Jentoft, Kaisorn Chaichana, Alfredo Quiñones-Hinojosa, R. Graham Cooks

**Affiliations:** 1 Department of Chemistry, Purdue Center for Cancer Research, and Bindley Bioscience Center, Purdue University, 560 Oval Drive, West Lafayette, IN 47907 USA; 2 Present Address: Department of Pathology and Immunology, Washington University School of Medicine, St. Louis, MO USA; 3 Department of Neurosurgery, Mayo Clinic, Jacksonville, FL USA; 4 Department of Radiology, Mayo Clinic, Jacksonville, FL USA; 5 Department of Laboratory Medicine and Pathology, Mayo Clinic, Jacksonville, FL USA

**Keywords:** Analytical chemistry, Bioanalytical chemistry, Mass spectrometry, Medical and clinical diagnostics

## Abstract

Tissue microarrays (TMAs) are commonly used for the rapid analysis of large numbers of tissue samples, often in morphological assessments but increasingly in spectroscopic analysis, where specific molecular markers are targeted via immunostaining. Here we report the use of an automated high-throughput system based on desorption electrospray ionization (DESI) mass spectrometry (MS) for the rapid generation and online analysis of high-density (6144 samples/array) TMAs, at rates better than 1 sample/second. Direct open-air analysis of tissue samples (hundreds of nanograms) not subjected to prior preparation, plus the ability to provide molecular characterization by tandem mass spectrometry (MS/MS), make this experiment versatile and applicable to both targeted and untargeted analysis in a label-free manner. These capabilities are demonstrated in a proof-of-concept study of frozen brain tissue biopsies where we showcase (i) a targeted MS/MS application aimed at identification of isocitrate dehydrogenase mutation in glioma samples and (ii) an untargeted MS tissue type classification using lipid profiles and correlation with tumor cell percentage estimates from histopathology. The small sample sizes and large sample numbers accessible with this methodology make for a powerful analytical system that facilitates the identification of molecular markers for later use in intraoperative applications to guide precision surgeries and ultimately improve patient outcomes.

## Introduction

Tissue microarrays (TMAs) are widely utilized tools for high-throughput pathology and molecular biology^1–6^. A TMA typically consists of hundreds of small tissue core samples, derived from many donor specimens, and embedded in a paraffin block^1–11^. Sectioning of a TMA then yields multiple slides containing hundreds of tissue sections (0.6–3 mm in diameter) arranged in a grid pattern that allows simultaneous examination of many clinical samples under identical conditions^1–6,9–11^. The use of TMAs has facilitated the rapid and efficient molecular profiling of tissue samples through parallel evaluation of one or several molecular markers at the protein, DNA, or RNA level, using techniques such as immunohistochemistry (IHC), fluorescence in situ hybridization, or mRNA in situ hybridization, respectively^1,3,5–7,10,12^. Nowadays TMAs are utilized to screen different tissue types, assess the clinical significance of molecular alterations in cancer and other diseases, and to evaluate the effectiveness of novel pharmaceuticals^12–24^. Such wide applications of TMAs follow from their clear advantages over individual tissue examination, which include decreased assay time and cost, experimental uniformity, and low sample utilization^1,4,6,8–10^.

In contrast to antibody-based IHC and in situ hybridization screens, label-free methods for TMA analysis allow for untargeted exploratory studies or rapid evaluation of specific biomarkers without use of staining protocols, providing further flexibility for the use of TMAs in clinical research. For instance, Fourier transform infrared (FTIR) spectroscopic analysis of TMAs has been utilized for histopathologic recognition of prostate and breast cancer^25–28^, oral squamous cell carcinoma^29^, and even cellular and acellular tissue constituents^28,30^, in most cases relying on sophisticated machine learning algorithms for data analysis^26,30^. Similar applications have been demonstrated using Raman spectroscopy^31–34^. In the case of mass spectrometry (MS), characterization of TMAs has been mainly done using matrix-assisted laser desorption ionization (MALDI) and has been heavily focused on protein analysis^35–38^. This approach has been successful at the characterization and subtyping of lung^39,40^ kidney^41^, and thyroid^42^ cancers, as well as the prediction of treatment response in breast cancer xenografts^43^ and in head and neck cancer samples^44^. Note that MALDI allows for the high-throughput analysis of TMAs, but it usually requires ionization in vacuum using a laser source and typically involves extensive sample preparation. Specifically for TMA analysis, widely reported MALDI protocols include TMA slide deparaffinization, heat-induced antigen retrieval, in situ enzymatic digestion, and matrix deposition steps before MS analysis^35,37,38^.

In this work we propose a simple method for the rapid generation of high-density (up to 6144 samples per array) TMAs followed by direct (i.e. no sample workup) online analysis using desorption electrospray ionization (DESI) MS. DESI is an ambient ionization technique for direct analysis of complex samples, such as tissue, in the open air^45–47^. It utilizes a charged solvent spray to impact the sample surface, desorb and ionize the molecules present, and carry them to the mass spectrometer for analysis^45^. As spatial control of the sampling event is readily available, DESI-MS has been extensively utilized for imaging of many complex biological samples, including various tissue types where the small-molecule profiles obtained have allowed for differentiation between cancerous and non-cancerous tissue in a multitude of human organs, albeit in a low-throughput manner^47–54^. Recently, high-throughput (> 1 sample per second) DESI-MS analysis has been successfully demonstrated for the screening of organic reactions^55–63^, the development of label-free biological assays^64,65^, and the generation of large spectral libraries^66,67^ using an automated platform that combines custom and commercial robotics, software, and MS instrumentation^68^. Here we expand the capabilities of this high-throughput DESI-MS platform to the rapid creation and direct characterization of TMAs via their small-molecule profiles using frozen brain biopsies as a proof-of-concept study. We anticipate this high-throughput methodology to have applications in retrospective studies and in support of MS-based intraoperative diagnosis^69–78^, the latter by aiding in the efficient identification of new biomarkers or the generation of robust classification models (both using large sample sets) which can then be translated to point-of-care applications in the operating room.

## Methods

### Samples

Two TMAs were analyzed in this study. The first TMA (TMA1) was composed of a collection of 36 frozen and unmodified human brain tissue biopsies purchased by Purdue University from the Biorepository of the Methodist Research Institute (Indianapolis, IN, USA). It is worth noting that this set of biopsies was acquired more than six years ago, sampled and analyzed on multiple occasions, and repeatedly freeze-thawed. However, it has an assortment of non-cancerous brain parenchyma samples, together with glioma, meningioma, and pituitary tumors, thus the choice to utilize it as part of this proof-of-concept study. The second TMA (TMA2) comprised 30 clinical human brain tissue biopsies from 9 patients undergoing tumor resection surgeries performed at Mayo Clinic (Jacksonville, FL, USA). The biopsies were taken from surgeon-defined positions within the tumor cavity with sampling positions being recorded by neuronavigational software. These biopsies were initially stored in the Biospecimen Accessioning and Processing Core at Mayo Clinic Florida and later sent to Purdue University for high-throughput DESI-MS analysis.

### Histopathological and genetic analysis

All tissue biopsies were subjected to pathologic evaluation. For the banked tissue samples (TMA1), tissue biopsies were cryosectioned, mounted onto glass slides, and hematoxylin and eosin (H&E) stained. For the samples in TMA2, fresh tissue biopsies were smeared onto glass slides and H&E stained. Evaluation of disease state, tumor cell percentage (TCP), glioma subtype, and tumor grade of all tissue biopsies were made by senior neuropathologists (TMA2: M.J.). The IDH genotype of each subject was confirmed by IHC and/or genetic testing of a pathologic biopsy, methods consistent with standard of care. All the de-identified pathological information is summarized in the Supporting Information (Supplementary Tables S1 and S2).

### Generation and high-throughput DESI-MS(/MS) analysis of TMAs

High-density tissue microarrays (up to 6144 samples/array) were generated using a Beckman Biomek i7 fluid handling workstation (Beckman Coulter, Indianapolis, IN, USA) equipped with a 50-nL floating slotted 384-pin tool (V&P Scientific, San Diego, CA, USA). Brain biopsies were placed in individual wells of a 384-well microtiter plate (Greiner Bio-One, Monroe, NC, USA) which was then located on the deck of the workstation. Minimal amounts of tissue (< 500 ng) were sampled simultaneously from the microplate using the pin tool and spotted onto a DESI slide generating sample spots of ca. 800 µm diameter. DESI slides are prepared in house using custom soda-lime glass slides with the same footprint as a well plate (Abrisa Technologies, Santa Paula, CA, USA) and coated with a porous PTFE membrane (Zytex G-115; Saint-Gobain, Wayne, NJ) using a light film of low VOC spray adhesive (Scotch Spray Mount; 3 M, St. Paul, MN). Up to 6144 samples (equivalent to 16 individual 384-well plates) can be spotted on a single DESI plate with a center-to-center distance of ca. 1.1 mm at the highest density. The spotting step is fast, flexible (i.e. not all positions have to be utilized), and allows transfer of replicates from the same tissue sample, thus providing a more accurate average molecular profile of the tissue within the analysis of a single array. In this work, each biopsy was spotted four times, generating a 1536-position grid (note that all available positions were not completely utilized on any TMA). Importantly, as only ng amounts of sample are transferred, the source plate containing the almost intact tissue biopsies can be easily frozen and stored for further analyses in a virtually unperturbed state. Note that depending on the state of the tissue, the quality of the spotted sample might vary. In particular, we noticed occasional incomplete transfers from older and repeatedly freeze-thawed samples as well as from tissue showing a high degree of coagulation or necrosis.

After spotting, the slides are automatically transferred to a mass spectrometer equipped with a DESI stage and analyzed in a *spot-to-spot* manner after calibration of sample positions using three reference dye-marks near the corners of the plate. The effective analysis time (i.e. time spent on each sample) is ca. 500 ms in full scan mode and about 6 s for tandem MS (MS/MS) analysis, enough to obtain high-quality spectra regardless of the inherent variability across the spotted samples (as displayed in Supplementary Figure S1), likely from differences in transfer efficiency and amount of blood present. Both the data acquisition and DESI stage movement are automatically controlled using custom Python software^59^. In this study, we chose to use two different mass spectrometers for TMA analysis. Full scan MS for untargeted molecular profiling of the tissue samples was carried out in the negative ion mode using a Synapt G2-Si quadrupole time-of-flight mass spectrometer equipped with a 2D DESI stage, a high-performance XS-generation DESI sprayer^79^, and a heated transfer capillary (Waters, Milford, MA, USA). The DESI spray solvent flow rate was set to 1.5 µL/min and the nebulizing nitrogen pressure was regulated to 20 psi. The heated transfer capillary was kept at 400 °C while the source temperature was set to 150 °C. Finally, the DESI spray voltage was selected as − 0.75 kV and the scan time was fixed at 100 ms. MS/MS analysis was also performed in the negative ion mode using an LTQ XL linear ion trap (Thermo Scientific, Waltham, MA, USA) equipped with a DESI 2D stage (Prosolia, Zionsville, IN, USA). In this case the DESI spray solvent flow was set to 3 µL/min and the nebulizing nitrogen pressure was fixed at 150 psi. The transfer capillary temperature was kept at 350 °C and the DESI spray voltage at − 4.5 kV. Automated gain control was utilized with a maximum injection time of 500 ms. Methanol Chromasolv LC–MS grade (Honeywell Riedel-de Haën, Muskegon, MI, USA) was used as DESI solvent in all cases.

Full scan MS analysis was carried out for the untargeted molecular profiling of the tissue samples in order to assess the potential of the small-molecule profile to differentiate tissue types or correlate with estimated TCP values. On the other hand, MS/MS analysis was utilized for a targeted study aimed at the rapid determination of isocitrate dehydrogenase (IDH) mutation status of the brain cancer samples. This approach follows previous studies^70,73,78^ that have demonstrated the excellent classification performance of IDH-mutant and IDH-wildtype gliomas using the ratio between 2-hydroxyglutatic acid (2HG, *m/z* 147), an oncometabolite accumulated in IDH-mutant tissue, and glutamic acid (Glu, *m/z* 146), an endogenous compound whose concentration is typically reduced in IDH-mutant tumors^80–84^. Thus, large 2HG:Glu ratios characterize IDH-mutant samples, whereas values close to zero are typical of IDH-wildtype tumors. To evaluate this ratio a single MS/MS experiment was utilized, simultaneously isolating both precursor ions using a window of *m/z* 3 units centered at *m/z* 146.5, then fragmenting both precursor ions simultaneously through collision-induced dissociation (CID; collision energy was set to 20 normalized manufacturer units), and evaluating their most intense transitions, i.e. *m/z* 147 → *m/z* 129 for 2HG and *m/z* 146 → *m/z* 128 for Glu. Note that the use of an ion ratio compensates for temporal variations in ion signal while the use of MS/MS provides higher specificity and reduced chemical noise.

**Figure 1:**
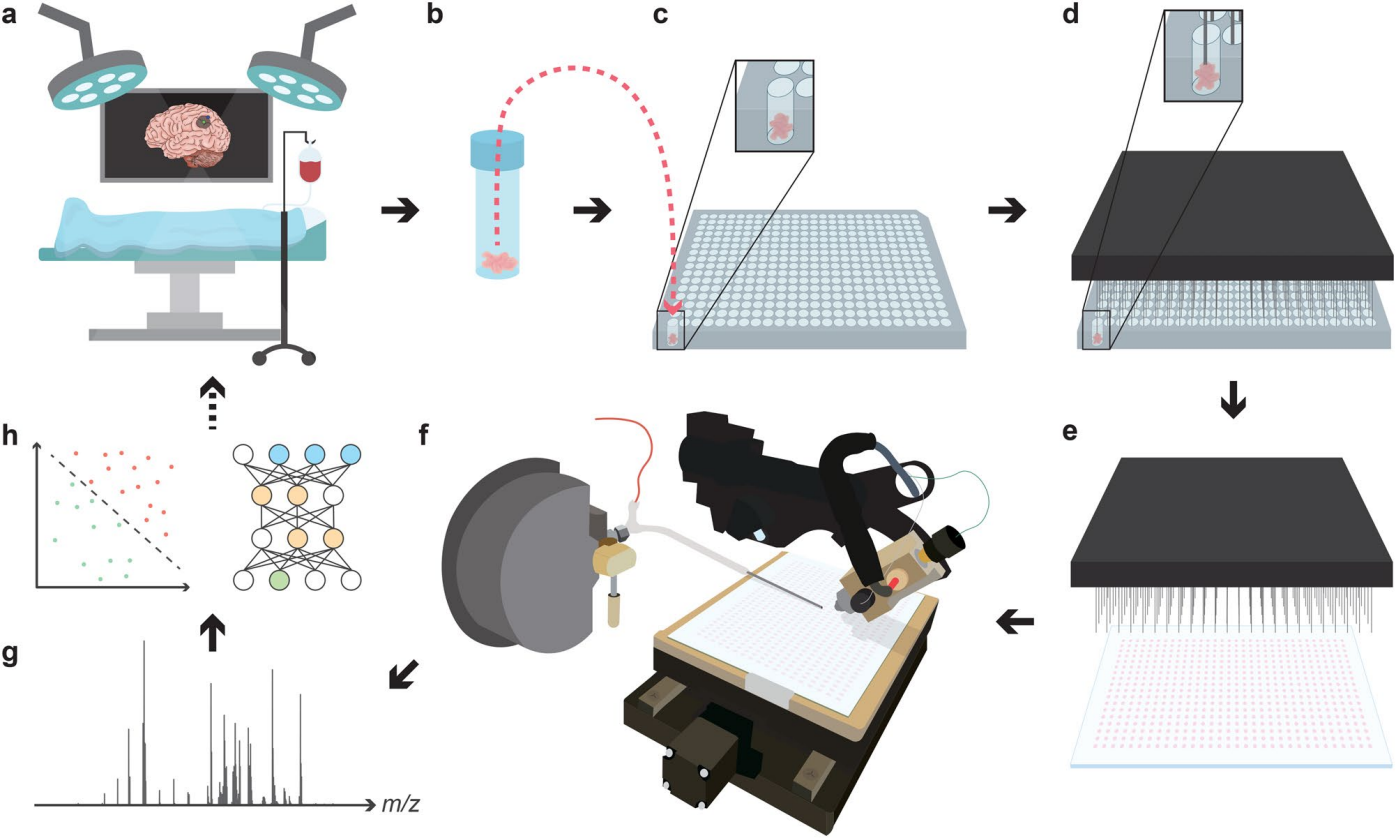
Overall workflow for the generation and high-throughput analysis of TMAs using automated DESI-MS. Biopsies are collected (**a, b**) and then arranged on a 384-well plate (**c**). Nanogram amounts of each biopsy are sampled using a pin-tool (**d**) and transferred to a DESI plate generating arrays with up to 6144 samples per plate (**e**). Both of these latter steps are carried out automatically using a fluid handling workstation. In this study, each biopsy was spotted four times to generate replicate samples in each TMA. Once ready, the plates are transferred to a DESI stage coupled to a mass spectrometer for analysis in a spot-to-spot fashion with throughputs better than 1 sample per second in full scan mode (**f**). Once the analysis is completed, spectra are automatically processed and assigned to the corresponding samples (**g**). Finally, the processed spectral data is utilized to identify/validate molecular markers or train classification methods (**h**) that can then be translated to MS-based intrasurgical applications.

The overall workflow is summarized in Fig. 1. Further details on hardware, software, and operation of the automated DESI-MS system have been described previously^59,68^.

### Data analysis

High-throughput DESI-MS full scan data for each array was acquired as a single RAW file whereas MS/MS spectra were recorded as one RAW file per each sample. In both cases, after acquisition was completed, RAW files were converted to mzXML format using MSConvert^85^ and automatically processed using custom MATLAB (MathWorks, Natick, MA, USA) scripts. Briefly, MS spectral data was assigned to each individual spot using the DESI stage log (i.e. time, position), and further assigned to biopsy samples using the source plate layout input by the user (i.e. which biopsy was located in each well). To obtain equally spaced spectral vectors with common separation-unit values, individual scans were resampled and averaged across each spot. Average spot spectra were normalized to the base peak and then used to calculate average results per biopsy.

Average full scan mass spectra were initially subjected to principal component analysis (PCA) after standard normal variate (SNV) normalization. The estimated principal components were utilized as input features for supervised classification using the classification learner toolkit in MATLAB. Due to the small sample size, especially when considering individual class representation, models were trained using six-fold cross-validation. Several common robust machine learning algorithms were explored, namely bagged and boosted trees, as well as linear support vector machine. Optimized hyperparameters, identified using Bayesian optimization, as well as further model details are described in the Supporting Information (Supplementary Table S3). Significant compounds were tentatively assigned using exact mass measurements (mass error < 5 ppm), previous literature reports on MS profiling of brain samples^54,71,86–91^, and the LIPID MAPS data base^92,93^.

MS/MS spectra were readily assigned to biopsy samples using the source plate layout as sample position information is recorded within the file name of each MS/MS RAW file. Similarly, as only single spots on the array were used for MS/MS analysis, the scans in the file were automatically averaged to yield the results for each biopsy. The intensities of both 2HG and Glu were extracted from these averages as 1 m*/z* unit wide bins centered on the product ion signals at *m/z* 129 and *m/z* 128, respectively. Finally, the IDH mutation score was calculated for each biopsy using the extracted ion intensity information. This score is simply computed as the ratio of 2HG and Glu intensities with a correction for the ^13^C-Glu isotope product ion contribution to the 2HG signal, as shown in Eq. (1).

1 $$IDH\,mutation\,score = \frac{{I_{129} - \left( {I_{128} \times 6.1\% } \right)}}{{I_{128} }}$$

### Ethical approval

Tumor and non-tumor tissue was obtained from patients undergoing craniotomies at Mayo Clinic Florida with approval of the Mayo Clinic Institutional Review Board (protocol 19-010725). Informed patient consent was obtained prior to collection. All methods were performed in accordance with relevant guidelines and regulations of the Institutional Review Board and the Declaration of Helsinki.

## Results and discussion

### IDH genotype assignment through targeted high-throughput DESI-MS/MS

**Figure 2:**
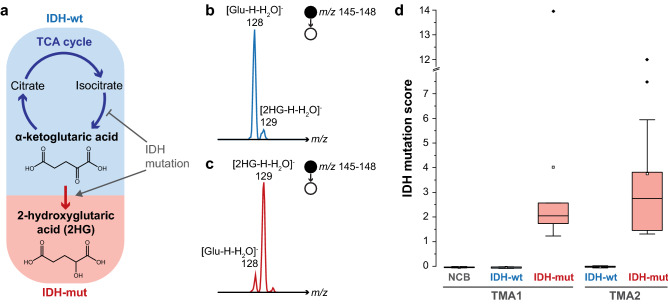
IDH genotype determination using high-throughput DESI-MS. In IDH-mutant (IDH-mut) gliomas, an alteration in the function of the IDH enzyme leads to the accumulation of 2HG (**a**), an oncometabolite that can be monitored easily by MS/MS in the negative ion mode. In a single MS/MS scan, using a 3 m*/z* unit window, both 2HG and Glu can be isolated and fragmented through CID allowing their [M–H–H_2_O]^−^ fragments to be monitored. Representative spectra for IDH-wildtype (IDH-wt; **b**) and IDH-mut (**c**) are both shown. Perfect classification of the samples in both TMAs (**d**) is achieved using the IDH mutation score (i.e. MS/MS ion ratio corrected for the ^13^C-Glu isotopic contribution) calculated from 6 s of MS/MS acquisition per sample. In all cases boxes show the 25th–75th percentile with the median, while whiskers correspond to 1.5 interquartile range (IQR); averages and outliers (outside 1.5 IQR) as white and black symbols, respectively. NCB: non-cancerous brain samples.

Molecular markers underlie the diagnosis, prognosis, and potential treatment of gliomas^94–96^. One such marker is associated with the mutation of the IDH enzyme, which plays a significant role in the accurate classification of gliomas and has prognostic value^97^, as patients with IDH-mutant gliomas survive longer^98,99^ and can benefit from extensive resection^100,101^. Mutation of this enzyme, whose wildtype function is the oxidative decarboxylation of isocitric acid to form α-ketoglutaric acid, leads to the accumulation of 2HG (Fig. 2a)^102,103^, which can be readily measured intraoperatively by MS/MS using biopsy smears in ca. 3 min per sample^70,73,78^. In laboratory settings, lower-throughput (ca. 30 min per sample) hyphenated chromatography-MS approaches have also been reported^104^. Here we evaluate a targeted MS/MS approach for high-throughput (6 s per sample) IDH genotyping of glioma using TMAs.

The proposed MS/MS assessment relies on the simultaneous isolation and fragmentation of 2HG and Glu for calculation of an IDH mutation score. Despite the rapid analysis and low sample amounts, high quality signal is obtained, as observed in the representative MS/MS spectra corresponding to IDH-mutant and IDH-wildtype biopsies shown in Fig. 2b, c. While only tumor core biopsies are used for the assessment of IDH genotype intraoperatively due to tumor heterogeneity, we analyzed all samples on the TMAs and compared with the pathological information. Even so, perfect classification performance (100% accuracy within the sample set) is obtained for differentiation of IDH-mutant samples (n_TMA1_ = 6; n_TMA2_ = 13) from IDH-wildtype (n_TMA1_ = 7; n_TMA2_ = 17) or non-cancerous brain biopsies (n_TMA1_ = 14) for both TMAs (Fig. 2d). Such high accuracy of the DESI-MS/MS based IDH genotyping suggests the ability of this high-throughput method to validate molecular diagnostic approaches using large banked sample sets.

### Untargeted metabolic profiling with high-throughput DESI-MS

**Figure 3:**
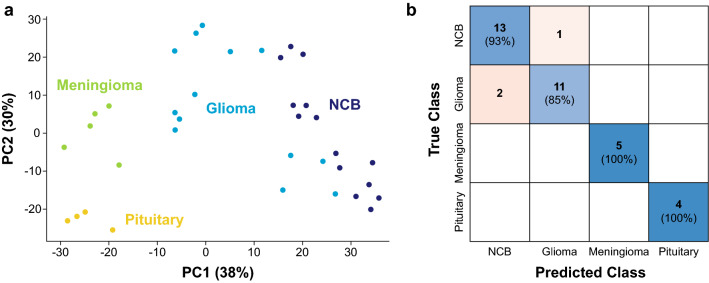
Untargeted analysis of TMA1 using full scan mass spectral data acquired in the negative ion mode. (**a**) Unsupervised clustering by tissue types was observed after PCA in the two-dimensional space generated with the first two principal components estimated (68% variance explained). Supervised classification of the samples using all the obtained principal components as features (m = 35) yielded overall 91.7% classification accuracy using a random forest model. (**b**) The model results are shown in the confusion matrix estimated via six-fold cross-validation. NCB: non-cancerous brain.

To assess the value of high-throughput DESI-MS for untargeted TMA metabolic profiling, we performed full MS analysis (1 s per sample) of both TMAs in the negative ion mode. In this case, the data from both arrays were analyzed separately as it has been demonstrated that the aging of biopsies, together with repeated freeze–thaw cycles^105^, induce metabolic alterations that largely dominate the variability amongst samples even in the case of formalin-fixed paraffin-embedded specimens^106^. PCA of all samples in TMA1 showed unsupervised clustering by tissue type in the three-dimensional space of the first two principal components (68% variance explained), with pituitary (n = 4) and meningioma (n = 5) tumors differing most from glioma (n = 13) and from non-cancerous brain parenchyma (n = 14) samples (Fig. 3a). These results are to be expected as the cells and tissues from which the different tumors arise are distinct (e.g. glia vs. meninges), and in agreement with previous studies^54,107^. In fact, using the estimated principal components as input features (m = 35), high accuracy (> 90%; ROC AUCs ≥ 0.89) is obtained for supervised tissue classification using simple machine learning algorithms including bagged (Fig. 3b) and boosted trees, as well as support vector machine (Supplementary Table S3). In most cases, as expected from visual examination of the PCA results, misclassifications tend to occur only between non-cancerous brain parenchyma and glioma samples, particularly those that seem molecularly similar in the PCA space (Supplementary Figure S2). Interestingly, the glioma samples commonly misclassified as non-cancerous brain across the algorithms tested, correspond all to low-grade (WHO grade II) gliomas, which tend to be diffuse and are characterized by a low-proliferation index^108,109^. This phenomenon clearly illustrates the complexity in classifying disease state in the presence of contributions by signals from both background parenchyma and tumor infiltration. Such tissue heterogeneity, especially near surgical margins, has been observed both intra- and post-operatively. Additional variability arising from glioma subtypes which might also affect classification performance has been previously reported^86,110–112^. This effect could not be probed due to the small sample size (n = 13 for all glioma samples; subtype class size: 1 ≤ n ≤ 5), but we anticipate that it could be assessed using the HT DESI-MS methodology described here together with larger banked sets.

**Figure 4:**
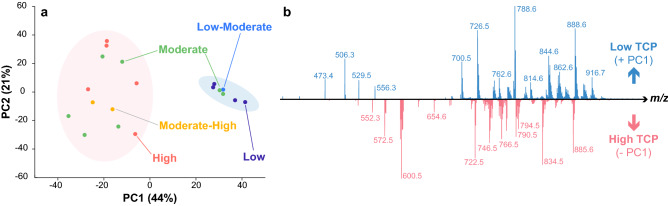
Untargeted analysis of TMA2 using full scan mass spectral data acquired in the negative ion mode. (**a**) Unsupervised clustering by estimated TCP was observed after PCA in the two-dimensional space generated with the first two principal components estimated (68% variance explained). (**b**) Observed separation between both groups is primarily associated with the first principal component (44% variance explained).

Unsupervised analysis of the results for TMA2, which contains only clinical glioma samples, agrees with previous reports on the correlation between the molecular profile of glioma biopsies and their estimated TCP^54,69,78^, as it shows two main clusters in the two-dimensional PCA space (65% explained variance), one of low and low-moderate TCP samples and the other corresponding to biopsies identified with high and moderate-high TCP (Fig. 4a). Samples labeled as moderate TCP are found distributed across both clusters. Interestingly, the observed separation between the two groups is primarily captured by the first principal component (44% variance explained), making straightforward the identification of relevant features that characterize high TCP samples vs. low TCP ones through the calculated coefficients of this component (Fig. 4b; Supplementary Table S4) as well as the average mass spectra corresponding to each cluster (Supplementary Figure S3). Relevant compounds previously identified^54,71^ as markers of non-cancerous gray and white matter, including phosphatidylserine (PS) 36:1 (*m/z* 788.5) and the sulfatides (STs) 24:1 (*m/z* 888.6) and 24:1(OH) (*m/z* 904.6), were found clearly associated with lower TCP samples. Similarly, phosphatidylethanolamine (PE) 38:4 (*m/z* 766.5), phosphatidylcholine (PC) 34:1 ([M + Cl]^−^; *m/z* 794.5), and phosphatidylinositol (PI) 38:4 (*m/z* 885.6), which have been previously identified with glioma tissue^54,71^, are associated with higher TCP biopsies. PS 40:6 (*m/z* 834.5), formerly observed in both non-cancerous and glioma samples^54^, was found more strongly associated with high TCP, as were PEs (40:6, *m/z* 790.5), plasmenyl-PEs (40:6, *m/z* 774.6; 38:4, *m/z* 750.5; 38:6, *m/z* 746.5; 36:4, *m/z* 722.5), and ceramides (40:2, *m/z* 654.6; 38:1, *m/z* 628.5; 36:1, *m/z* 600.5; 36:2, *m/z* 598.5; 34:1, *m/z* 572.5), these latter observed as [M + Cl]^−^ adducts. Similarly, additional molecules suggestive of low TCP, included STs (26:1, *m/z* 916.7; 22:0, *m/z* 862.6; 22:1, *m/z* 860.6; 18:0, *m/z* 806.5) and plasmenyl-PEs (38:2, *m/z* 754.6; 36.2, *m/z* 726.5; 34:1, *m/z* 700.5).

The results obtained through this rapid untargeted profiling experiment indicate that HT DESI-MS has potential to efficiently generate and validate models of infiltrative margin determination, which is still a significant challenge during tumor resection^100,111,113,114^. Such models hold high value as they could be directly translated to intraoperative MS diagnosis, an approach that has been increasingly demonstrated using both standard^69–71,74–78^ and miniature^72,73,115^ MS instrumentation, as well as MS coupled to robotic surgical systems^76^.

## Conclusions

DESI-MS is a common method for imaging of tissue sections and has been widely utilized to characterize multiple disease states in many tissue types. However, imaging typically requires long analysis times that are not compatible with high-throughput evaluation of large sample sets, such as TMAs. Here we demonstrate a novel application of an automated high-throughput DESI-MS platform to generate and directly analyze high-density TMAs from frozen, unmodified tissue biopsies. Using a robotic fluid-handling workstation equipped with a pin tool we can create TMAs with up to 6144 samples per array using sub-microgram amounts of tissue. These TMAs are then automatically analyzed using DESI-MS with throughputs better than 1 sample per second for full scan MS screening and at rates close to 6 s per sample for MS/MS analysis. We demonstrated this new approach using brain cancer samples, showing that despite the low sample usage and the short analysis time, the spectral data obtained is of high quality and in agreement with previously reported results. In particular, we were able to assess IDH genotypes with 100% accuracy using a targeted MS/MS approach, clearly differentiating IDH-mutant from IDH-wildtype or non-cancerous brain parenchyma samples in two TMAs. We also explored the potential of using MS spectra as molecular profiles of the biopsies to differentiate tissue type (non-cancerous parenchyma, glioma, meningioma, pituitary) or to correlate with estimated TCP. Considering its minimal sample preparation, small volume of sample required, and high throughput, automated high-throughput DESI-MS also has potential value for the generation of spectral libraries for sample classification, the identification of biomarkers through large-scale studies, and the testing for biochemical activity or drug distributions in tissue.

## Supplementary Information

Supplementary Information.

## Data Availability

The raw datasets generated during and analyzed during the current study are available from the corresponding author on reasonable request.
