# Supplementary material for: High-throughput analysis of tissue microarrays using automated desorption electrospray ionization mass spectrometry
Source: Sci Rep. 2022 Nov 7;12:18851. doi: 10.1038/s41598-022-22924-4 (PMC9640715; doi:10.1038/s41598-022-22924-4)
Supplement: Supplementary file 1 — Supplementary Information. [file 41598_2022_22924_MOESM1_ESM.pdf]

Supplementary Information

High-Throughput Analysis of Tissue Microarrays using Automated Desorption Electrospray Ionization Mass Spectrometry

Nicolás M. Morato,[1†] Hannah Marie Brown,[1†‡] Diogo Garcia,[2] Erik H. Middlebrooks,[2,3] Mark Jentoft,[4] Kaisorn Chaichana,[2] Alfredo Quiñones-Hinojosa,[2] and R. Graham Cooks[1]*

[1] Department of Chemistry, Purdue Center for Cancer Research, and Bindley Bioscience Center, Purdue University, West Lafayette, IN, USA
[2] Department of Neurosurgery, Mayo Clinic, Jacksonville, FL, USA
[3] Department of Radiology, Mayo Clinic, Jacksonville, FL, USA
[4] Department of Laboratory Medicine and Pathology, Mayo Clinic, Jacksonville, FL, USA

† *Authors contributed equally*

‡ Current affiliation: Department of Pathology and Immunology, Washington University School of Medicine, St. Louis, MO, USA

**Corresponding author:*
R. Graham Cooks
cooks@purdue.edu
560 Oval Drive, Department of Chemistry, Purdue University, West Lafayette, IN 47907, USA

Supplementary Table S1. De-identified histopathological information of the TMA1 samples. Sample numbers reference our internal tissue bank indexing and are consistent throughout the Supplementary Information. All samples described here were subjected to untargeted MS analysis whereas only those with IDH genotype information, together with *non-cancerous brain* biopsies, were analyzed through MS/MS.

Sample	Diagnosis	Comments	IDH Genotype
1	Anaplastic astrocytoma	WHO grade III	IDH-1 immunoreactive
2	Non-cancerous brain	-	-
3	Non-cancerous brain	-	-
4	Non-cancerous brain	-	-
5	Non-cancerous brain	-	-
6	Non-cancerous brain	-	-
7	Non-cancerous brain	-	-
8	Non-cancerous brain	-	-
10	Non-cancerous brain	-	-
11	Non-cancerous brain	-	-
12	Non-cancerous brain	-	-
14	Non-cancerous brain	From GBM patient	-
15	Non-cancerous brain	From GBM patient	-
16	Non-cancerous brain	From GBM patient	-
17	Non-cancerous brain	From GBM patient	-
20	GBM	-	IDH-1 non-immunoreactive
21	GBM	-	IDH-1 non-immunoreactive
22	GBM	WHO grade IV	IDH-1 non-immunoreactive
23	GBM	WHO grade IV; recurrent/residual	IDH-1 non-immunoreactive
24	GBM	WHO grade IV; foci of oligodendroglial pattern; deletion 1p36 not identified; deletion 19q13 not identified	IDH-1 non-immunoreactive
26	Meningioma	-	-
27	Meningioma	-	-
28	Meningioma	-	-
30	Meningioma	-	-
31	Meningioma	-	-
32	Oligoastrocytoma	WHO grade III; anaplastic; recurrent	IDH-1 immunoreactive
33	Oligoastrocytoma	WHO grade II; negative for the 1p/19q co-deletion	IDH-1 immunoreactive
34	Oligoastrocytoma	WHO grade II; deletion 1p36 not identified; deletion 19q13 not identified	IDH-1 immunoreactive
35	Oligoastrocytoma	At least WHO grade II; recurrent	IDH-1 immunoreactive
37	Oligodendroglioma	WHO grade II; bulk of the tumor is made up of a low-grade oligodendroglial population	IDH-1 non-immunoreactive
39	Oligodendroglioma	WHO grade III; anaplastic; recurrent/residual	IDH-1 strongly immunoreactive
41	Oligodendroglioma	WHO grade III; anaplastic; deletion 1p36 not identified; deletion 19q13 not identified	IDH-1 non-immunoreactive
42	Pituitary	-	-
43	Pituitary	-	-
44	Pituitary	-	-
45	Pituitary	-	-

Supplementary Table S2. De-identified histopathological information of the TMA2 samples. Sample numbers reference our internal tissue bank indexing and are consistent throughout the Supplementary Information. All samples described here were subjected to targeted MS/MS analysis for IDH genotype determination. Only samples with TCP estimates were included in the untargeted MS data analysis.

Sample	Patient	Location	IDH Genotype	Pathology	TCP estimation
4	1	Margin	Wildtype	Non-cancerous brain	-
5		Core	Wildtype	Glioma	Low
7	2	Core	Mutant	Glioma	Moderate
8		Core	Mutant	Glioma	Moderate
9		Core	Mutant	Glioma	Moderate
10		Core	Mutant	Glioma	Moderate
13	3	Core	Mutant	Glioma	High
15		Core	Mutant	Glioma	Moderate
16	4	Margin	Wildtype	Glioma	High
18		Core	Wildtype	Predominantly blood; small glioma clumps	-
19		Margin	Wildtype	Predominantly blood; small glioma clumps	-
20	5	Margin	Mutant	Glioma	Low
22		Core	Mutant	Glioma	Low-moderate
23		Core	Mutant	Glioma	Moderate
25	6	Margin	Mutant	Glioma	Low
26		Margin	Mutant	Glioma	Low
28		Core	Mutant	Glioma	Moderate-high
29		Core	Mutant	Glioma	Moderate-high
30	7	Core	Wildtype	Glioma	High
32*		-	Wildtype	-	-
33*		-	Wildtype	-	-
34*		-	Wildtype	-	-
35*		-	Wildtype	-	-
36		Core	Wildtype	Glioma	High
37		Core	Wildtype	Glioma	High
39	8	Core	Wildtype	Glioma	Moderate
40		Core	Wildtype	Predominantly blood; high-cellularity clumps	-
47*	9	-	Wildtype	-	-
48*		-	Wildtype	-	-
49*		-	Wildtype	-	-

*Location information not recorded when biopsy was submitted to biobank, rendering it impossible to correlate pathological results with the biopsy.

Supplementary Table S3. Details on machine learning models trained and validated for supervised classification of tissue types using MS spectral information acquired in the negative ion mode. All models were optimized using Bayesian optimization (30 iterations) and the expected improvement per second as acquisition function. NCB: non-cancerous brain.

Model	Optimized hyperparameters	Performance
Bagged trees	Maximum number of splits: 35<br>Number of learners: 22<br>Number of predictors to sample: 35	Accuracy (validation): 91.7%<br>ROC AUC range (across classes; 1-vs-all): 0.89-1<br><br>See validation confusion matrix in **Figure 3B**
Support vector machine	Kernel function: Linear<br>Box-constraint level: 1.01<br>Multiclass method: One-vs-One<br>No data standardization	Accuracy (validation): 91.7%<br>ROC AUC range (across classes; 1-vs-all): 0.98-1<br>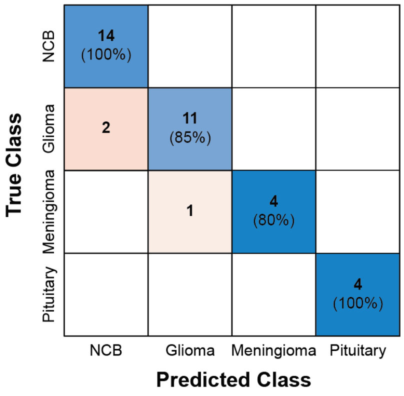
Boosted trees	Ensemble method: AdaBoost<br>Number of learners: 14<br>Learning rate: 0.39<br>Maximum number of splits: 3	Accuracy (validation): 91.7%<br>ROC AUC range (across classes; 1-vs-all): 0.93-1<br>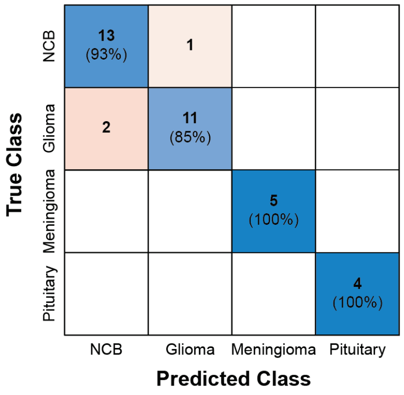

Supplementary Table S4. Compounds identified as relevant for the discrimination of low and high TCP samples. Low TCP samples were associated with positive coefficients on the first principal component (PC1). Coefficients were obtained upon PCA of the negative ion mode spectral data corresponding to TMA2.

Coefficient PC1	Measured mass	Expected mass	Mass error	Tentative identity
-0.12	572.4791	572.4809	-3.1	Cer 34:1*
-0.13	598.4970	598.4966	0.7	Cer 36:2*
-0.25	600.5093	600.5122	-4.8	Cer 36:1*
-0.02	628.5415	628.5435	-3.2	Cer 38:1*
-0.04	654.5621	654.5592	4.4	Cer 40:2*
-0.02	682.5895	682.5905	-1.5	Cer 42:2*
0.12	700.5275	700.5281	-0.9	pPE 34:1
-0.19	722.5121	722.5125	-0.6	pPE 36:4
0.22	726.5443	726.5438	0.7	pPE 36:2
-0.14	746.5123	746.5125	-0.3	pPE 38:6
-0.06	750.5430	750.5438	-1.1	pPE 38:4
0.06	754.5735	754.5751	-2.1	pPE 38:2
-0.13	766.5409	766.5387	2.9	PE 38:4
-0.10	774.5416	774.5438	-2.8	pPE 40:6
0.29	778.5443	778.5442	0.1	PS 36:1
-0.12	790.5393	790.5387	0.8	PE 40:6
-0.09	794.5447	794.5467	-2.5	PC 34:1*
0.04	806.5011	806.4972	4.8	ST 18:0
0.05	814.5558	814.5598	-4.9	PS 38:2
-0.21	834.5280	834.5285	-0.6	PS 40:6
0.14	844.6052	844.6068	-1.9	PS 40:1
0.07	860.5962	860.5921	4.8	ST 22:1
0.09	862.6100	862.6078	2.6	ST 22:0
-0.17	885.5497	885.5493	0.5	PI 38:4
0.22	888.6003	888.5966	4.2	ST 24:1
0.04	904.6156	904.6184	-3.1	ST 24:1(OH)
0.07	916.6564	916.6548	1.7	ST 26:1

*Exact expected masses calculated as $[M+^{35}Cl]^-$ adducts.

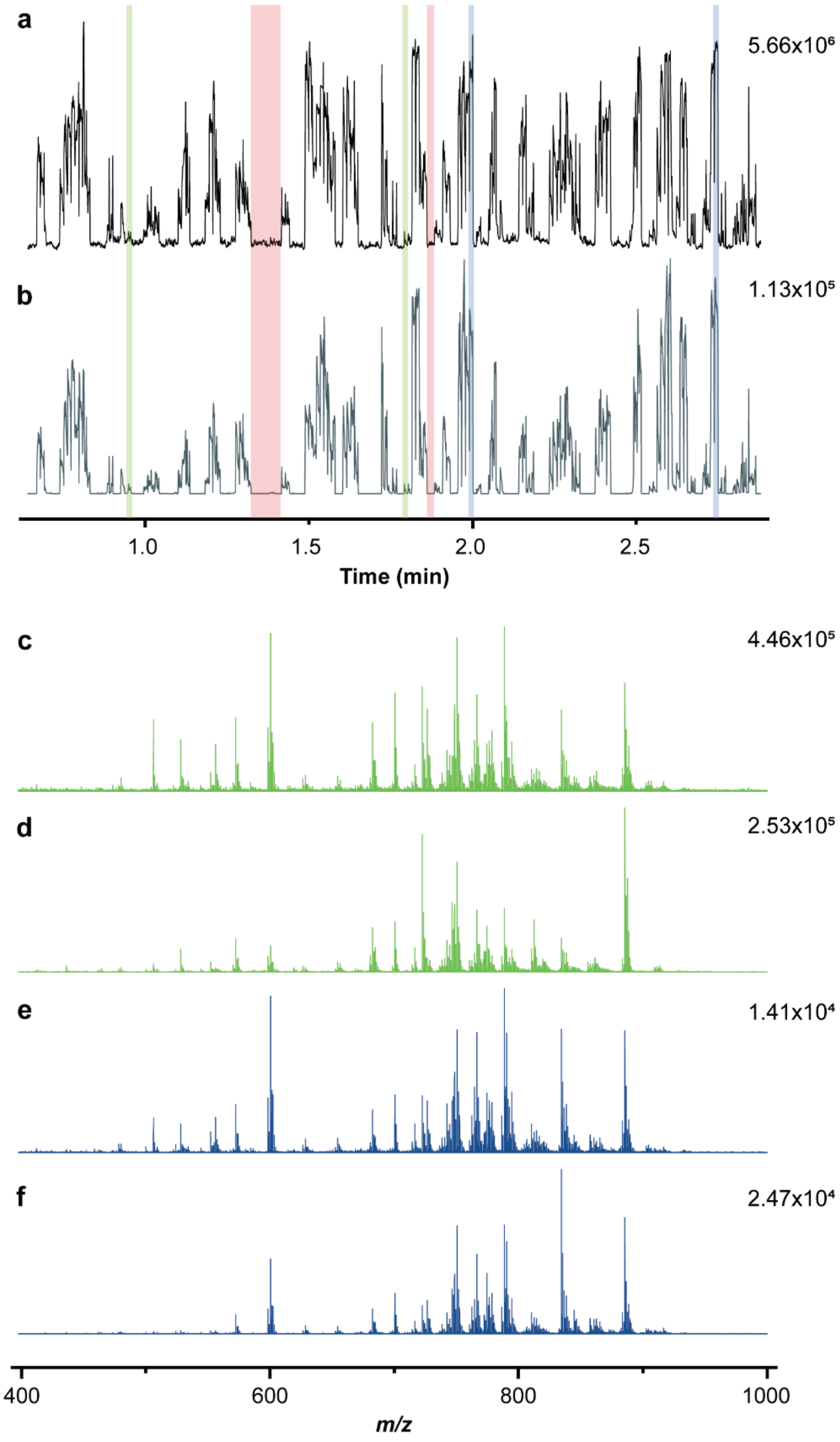

Supplementary Figure S1. Raw data results obtained by high-throughput DESI-MS analysis of a TMA in the negative ion mode. A section of the total ion chronogram (TIC, **a**) and the extracted ion chronogram for *m/z* 885.4 - 885.8 (**b**) are both shown to exemplify the raw intensity variability across samples, which is within an order of magnitude. Note that not all the wells on the biopsy master plate were occupied and their corresponding empty spots in the high-density TMA are observed when the signal drops to base line levels (sections highlighted in red are examples). Importantly, signal variability does not affect data quality as high signal-to-noise spectra are obtained from spots with both low (**c**, **d**) and high (**e**, **f**) overall signal. The spots from where these representative spectra were extracted are highlighted in blue (high intensity) and green (low intensity) in the ion chronograms. For the sake of easy visualization all the plots are normalized, however raw intensities are denoted in the top right corner of all the graphs.

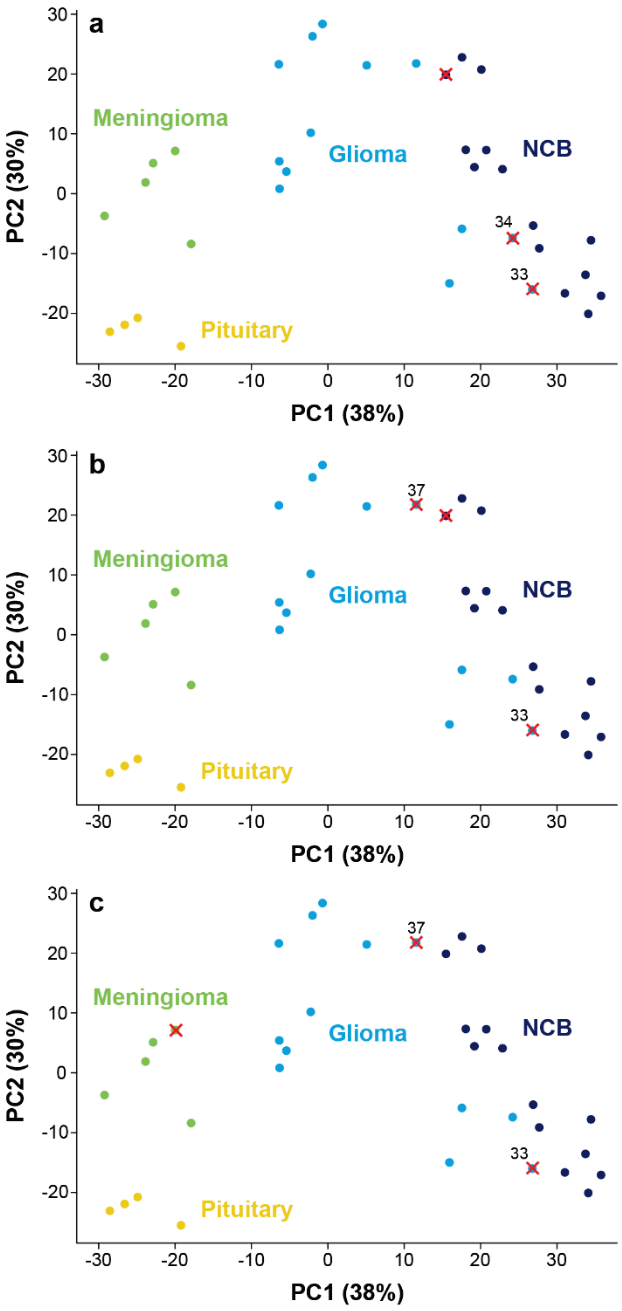

Supplementary Figure S2. Misclassifications observed using the bagged trees (**a**), boosted trees (**b**), and support vector machine (**c**) models. Three glioma samples often misclassified as non-cancerous brain (*33*, *34*, *37*), which can be observed to be molecularly similar in the 2D PCA space, are all low-grade gliomas (see **Table S1**). NCB: non-cancerous brain.

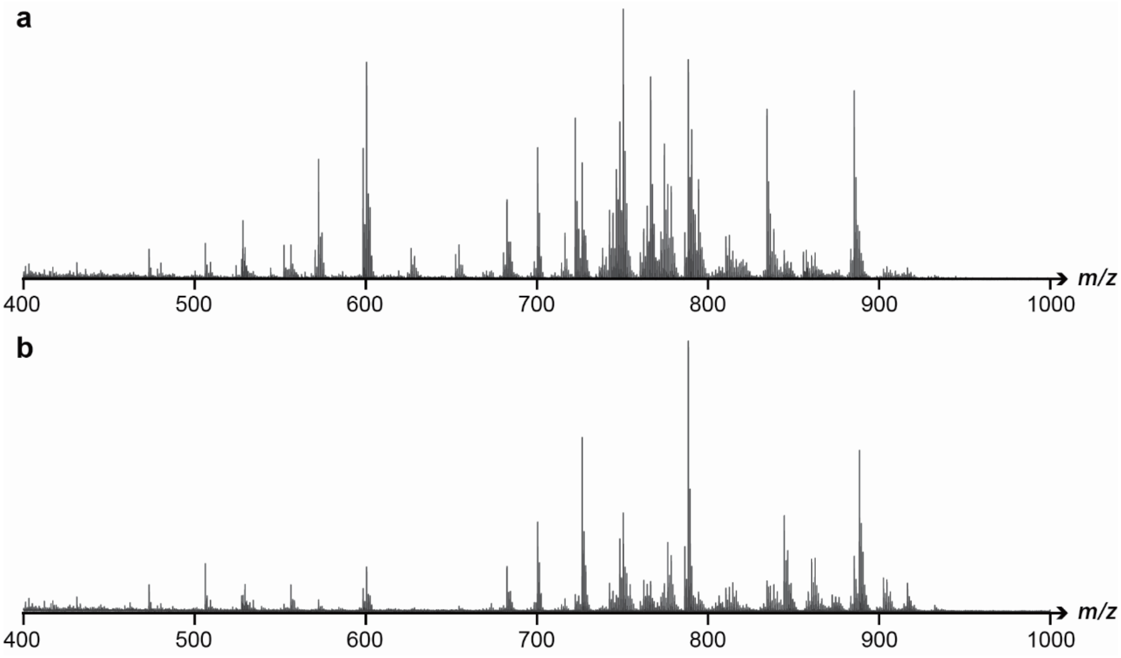

Supplementary Figure S3. Average negative ion mode mass spectra corresponding to the samples in TMA2 clustered as high (**a**) and low (**b**) TCP (as indicated by the histopathological estimates).
